# A time-robust group recommender for featured comments on news platforms

**DOI:** 10.3389/fdata.2024.1399739

**Published:** 2024-05-21

**Authors:** Cedric Waterschoot, Antal van den Bosch

**Affiliations:** ^1^KNAW Meertens Instituut, Amsterdam, Netherlands; ^2^Department for Language, Literature and Communication, Institute for Language Sciences, Utrecht University, Utrecht, Netherlands

**Keywords:** natural language processing, news recommendation, content moderation, online discussions, ranking

## Abstract

**Introduction:**

Recently, content moderators on news platforms face the challenging task to select high-quality comments to feature on the webpage, a manual and time-consuming task exacerbated by platform growth. This paper introduces a group recommender system based on classifiers to aid moderators in this selection process.

**Methods:**

Utilizing data from a Dutch news platform, we demonstrate that integrating comment data with user history and contextual relevance yields high ranking scores. To evaluate our models, we created realistic evaluation scenarios based on unseen online discussions from both 2020 and 2023, replicating changing news cycles and platform growth.

**Results:**

We demonstrate that our best-performing models maintain their ranking performance even when article topics change, achieving an optimum mean NDCG@5 of 0.89.

**Discussion:**

The expert evaluation by platform-employed moderators underscores the subjectivity inherent in moderation practices, emphasizing the value of recommending comments over classification. Our research contributes to the advancement of (semi-)automated content moderation and the understanding of deliberation quality assessment in online discourse.

## 1 Introduction

Online news platforms allowing user-generated comments have been facing challenges in terms of content moderation due to the ever-increasing content stream (Meier et al., 2018; Wintterlin et al., 2020). Discussions are increasing in size and toxicity, described under the term “dark participation,” is omnipresent (Quandt, 2018). The set of tasks assigned to the moderator has expanded, as well as the need to swiftly make difficult, interpretative moderation decisions (Paasch-Colberg and Strippel, 2022). Platforms are increasingly interested in computational solutions to aid human moderators in tasks such as filtering out toxicity, countering misinformation, and promoting high-quality user comments (Gollatz et al., 2018; Gillespie, 2020). Broadly speaking, content moderation strategies revolve around two main approaches: maintaining a comment space free of toxic and unwanted content, and recently, highlighting what platforms consider as “good” contributions, such as featuring them prominently on the webpage (Diakopoulos, 2015a; Roberts, 2017; Wang and Diakopoulos, 2022). However, manually selecting comments to feature is labor-intensive and demands substantial attention and resources from editorial staff and content moderators. To address this issue, we propose a group recommender system capable of recommending a set of qualifying comments, potentially streamlining the decision-making process.

We introduce classifiers designed to rank comments based on class probability, aiding comment moderators in selecting featured comments. Using Dutch comment data with human labeling of featured comments, we train a series of models which present the human moderator with curated comments deemed qualified to be featured. These models, referred to as group recommenders, are not personalized for each moderator but instead represent the moderation strategy for content moderators as a collective entity.

Our contribution adds to the ongoing research on (semi-)automated content moderation and the evaluation of deliberation quality. We achieve this by training and examining a range of classifiers and by creating practical evaluation scenarios that mirror the real-world process of selecting individual comments based on online discussions and their context. In practical terms, we depart from evaluating on artificially split or balanced datasets and instead assess our models on unseen discussion articles spanning both 2020 and 2023. This approach mirrors the evolving news cycles and platform growth over time. Our findings indicate that our best-performing models maintain their ranking performance even on recent articles. The final step in our realistic evaluation scenario is performed by moderators currently employed at the platform in question. Their expert evaluation highlights the subjectivity inherent in the practice, thereby reinforcing the argument in favor of recommending comments rather than solely relying on classification.

## 2 Materials and methods

### 2.1 Background

The commenting environments on online news platforms and their user bases have been growing, driving content moderators to adapt and expand their moderation strategies. Studying user participation, and the moderation of this content, has grown into an important focus of digital journalism scholars (Gillespie et al., 2020; Quandt, 2023). Initially, moderating online comments was focused on assessing the appropriateness of the comment in relation to the platform (Roberts, 2017; Gillespie, 2018). Dealing with such negative content has been a particular focus, e.g. (organized) misinformation campaigns (Meier et al., 2018; Zareie and Sakellariou, 2021) or online harassment (Quandt et al., 2022). Aside from such clear cases of toxicity, moderators were also tasked with dealing with gray cases, requiring a closer look at the perception of online incivility and hate comments (Paasch-Colberg and Strippel, 2022). Online activity of this form has been described under the term “dark participation” by Quandt (2018). Recently, however, a novel strategy emerged entailing the promotion of *high-quality* comments by content moderators (Wang and Diakopoulos, 2022). In an attempt to counteract dark participation, moderators are selecting what they deem good, feature-worthy comments, and are flagging them to be moved (pinned) to the top of the comment space.

Outside the context of online news platforms and their moderation strategies, deliberation quality has been widely studied (Friess and Eilders, 2015). However, it remains a struggle to define deliberation in diverse contexts (Jonsson and Åström, 2014). The featuring of individual comments by content moderators may be seen as an operationalization of the concept in one specific context, purely based on the interpretation by the moderators and guidelines set by the platform.

Looking at news outlets specifically, many platforms have been promoting what they see as high-quality contributions in recent years, for example in the form of New York Times (NYT) picks (Wang and Diakopoulos, 2022), Guardian Picks at the Guardian or featured comments at Dutch news outlet NU.nl (NUJij, 2018). Their FAQ pages describe such promotion-worthy comments as “substantiated”, “representing a range of viewpoints” or “respectful”^1^, ^2^. Previous research has termed such efforts as *empowerment moderation*, an attempt to motivate the user base to discuss in a constructive manner (Heinbach et al., 2022). The authors concluded that these efforts did decrease perceived toxicity on online news platforms. Ziegele et al. (2020) link news value theory to deliberative factors found in the comments posted on news articles, studying how particular characteristics of news articles influence the deliberative quality of social media comments replying to the news article. Diakopoulos (2015b) assigns a set of editorial criteria to featured comments on a news platform, in this case NYT picks. These range from argumentativeness to relevance to the discussion and entertainment value (Diakopoulos, 2015b). Generally, this moderation practice can be seen as a “norm-setting strategy” (Wang and Diakopoulos, 2022, p. 4). Supplementary to the goal of promoting high-quality user-generated content and the positive normative effect that it may have on others, user engagement might increase as well. Wang and Diakopoulos (2022) observe that users who received their first featured comment subsequently increased their own comment frequency.

Our task of ranking featured comments within online discussions is rather novel, but it is adjacent to the line of research on news recommendation. However, the task of news recommendation often entails personalization aimed at readers on news platforms (Raza and Ding, 2022). It differs from our application in that ours is aimed at improving the experience of the content moderators as opposed to that of the readers. In other words, our application supports the practice of content moderation, while news recommendation optimizes news consumption (Raza and Ding, 2022). Another adjacent field of research combining the use of Natural Language Processing (NLP) and online discussion and deliberation is argument mining (Lawrence and Reed, 2020). Aside from argumentative structure in online text samples, such applications have looked at possibilities to foster mutual understanding among discussion platform users and the evolution of quality deliberation among participants (Shin and Rask, 2021; Waterschoot et al., 2022). For example, previous research has used time series data to model the evolution of deliberation quality or adapters to model different quality dimensions (Shin and Rask, 2021; Falk and Lapesa, 2023). Building further on the usage of adapters for deliberation quality evaluation, Behrendt et al. (2024) combine both expert and non-expert labeling, using the correlation between the two categories to derive a singular quality measurement. Our task differs from these applications as this study does not focus on argumentation as an indicator for deliberation quality or due to the fact that we do not aim to construct a metric for assessing comment quality as a general concept. In this study, we take the historical moderation choices as the standard of what constitutes, through the lens of a specific online platform, a quality comment. Additionally, as opposed to the mentioned applications, our framework includes comment and user information alongside text representation.

Hybrid moderation is the result of moderators at online news outlets increasingly working with computational systems to execute their tasks (Gorwa et al., 2020; Lai et al., 2022). The hybrid nature is causing the role of human moderator on the one hand, and the computational system on the other to be intertwined (Gillespie, 2020). The goal of this approach is to make use of the strengths of both automated and manual content moderation (Jiang et al., 2023). Ideally, editors and moderators alike see the function of AI as offering decision support, instead of decision-making (Ruckenstein and Turunen, 2020; Jiang et al., 2023). This AI assistance has also been referred to as a “machine-in-the-loop” approach, elevating the human moderator to the central actor (Li and Chau, 2023). Such support for the moderator in executing their tasks allows the moderators themselves to adapt to the nuances and rapid changes in online contexts (Park et al., 2016). In as much as AI could save time, moderators are able to invest the nuanced human interpretation and judgement that certain edge cases require (Jiang et al., 2023). The strength of the automated half of the hybrid pipeline is the quantity of comments that can be moderated, especially in terms of clear-cut decisions (Jiang et al., 2023). Similar AI-assisted applications have been pursued on other types of online platforms, such as question answering platforms (Annamoradnejad et al., 2022) and social media platforms (Morrow et al., 2022).

Automatically detecting toxicity in online comment sections has received substantial attention (Gorwa et al., 2020; Wang, 2021). The classification of featured comments specifically, however, has not been explored quite that often and has remained understudied. Diakopoulos (2015a) uses cosine similarity to calculate article and conversation relevance scores using New York Times editor picks. The study concludes that such relevance scores are associated with New York Times picks and computational assistance based on such scoring may speed up comment curation (Diakopoulos, 2015a). Park et al. (2016) present their CommentIQ interface, which entails a Support Vector Machine (SVM) classifier on unbalanced, but limited, data (94 NYT picks, 1, 574 non-NYT picks). The included classifier achieves a precision score of 0.13 and recall of 0.60. Their dataset includes both user features as well as comment-specific variables (Park et al., 2016).

Napoles et al. (2017) present their ERICs framework annotating Yahoo News comments in terms of “Engaging, Respectful, and/or Informative Conversations.” Their work looks at constructive discussion at the thread level as opposed to singular comments. Additionally, their labeling is not based on editorial choices, as is the case for our featured comments or studies working with New York Times picks (Napoles et al., 2017). Kolhatkar and Taboada (2017) supplement those Yahoo comments with NYT picks. Using these picks as benchmark of constructive discussion, the authors achieve an F1-score of 0.81 using a BiLSTM on GloVe embeddings and a balanced training and testing set (Kolhatkar and Taboada, 2017). Furthermore, the study combines a large set of variables, including comment length features and names entities, to train SVMs which reach an F1-score of 0.84 on balanced sets (Kolhatkar and Taboada, 2017). In a follow-up study, the authors employed crowdsourced annotations and logistic regression to construct a similar tasks, yielding an F1-score of 0.87 (Kolhatkar et al., 2023).

In sum, classification of high-quality comments such as those featured by moderators is a task that has been explored relatively little. Aside from Park et al. (2016), classifiers proposed in earlier work lacked information outside comment content features, and focused on text representations or other comment features. Additionally, the validation of these models was performed on balanced test sets, which does not resemble the real-life practice of picking a few featured comments out of a discussion of a news article. The online content moderator chooses editor picks on the article level and, therefore, any model should be evaluated on this exercise. In this paper, we aim to address this practice by putting together all information available to the moderator while they perform their tasks, including user information, comment statistics and text representation. Next, we replicate the task of picking a few featured comments out of many at the level of the discussion of a news article as an evaluation of our models.

### 2.2 Platform specifics

The comment platform used in the current study is NUjij. This online reaction platform is part of the Dutch online newspaper NU.nl^3^. NUjij, which translates to “now you,” allows users to comment on a wide range of news articles published by the news outlet. Pre-moderation is set in place, consisting of automatic filtering of toxic content alongside the human moderators who check the uncertain comments (Van Hoek, 2020). The platform has a moderation pipeline that includes multiple strategies, including awarding expert labels to select verified users and pinning featured comments at the top of the comment section (NU.nl, 2020). As said, the latter is a moderation strategy also practiced at e.g. the New York Times or the Guardian.

Featured comments are chosen manually by the moderators at the platform. A comment is either featured or not. They define such comments as “substantiated and respectful” and “contributing to constrictive discussion”^4^. The FAQ page informs users that moderators are aiming to present a balanced selection of featured comments in terms of perspectives and to not pick based on political affiliations. This study addresses the specific issue of picking featured comments using the information available to the human moderators while they perform their tasks. These variables include user information and their commenting history, for example whether their comments have been featured before. While highlighting quality content in the form of featured comments is a common moderation practice, other platforms might have different editorial guidelines in place. To best support the moderator in efficiently featuring comments they deem worthy of the status, it is vital that the computational approach is fully suited to their specific platform and context. This may include the intended human bias in choosing such comments. Therefore, we aim to train models that rank comments replicating the choices made by NUjij moderators in the past.

### 2.3 Datasets and data splits

The current study includes two datasets from the Dutch platform, the first one containing articles from 2020 and the second originating from 2023. Each dataset consists of a single file containing observations on the comment level. Each comment is timestamped and has a user and article ID number. Additionally, each comment has information on whether it was rejected by a moderator, whether it was featured, the number of replies, the number of likes and the actual comment text. To mimic moderators' reading behavior, we discarded all comments within a discussion published after the timestamp of the final featured comment in that specific discussion. This pre-processing step contributes to mimicking the real-life practice including the time constraints content moderators deal with. The procedure of featuring comments takes place as the discussion is growing as opposed to dwindling down. There is no upper limit for featuring comments within a single discussion. Thus, including comments published later on in the discussion would produce results inconsistent with the practical nature of the content moderation process.

This procedure mimics the time-related nature of picking featured comments. The moderator performs this task in the earlier phases of the discussion to present users with the featured content while they are still participating.

Using the article ID, we scraped the topic of each article from the original web page. Each news article is given topical keywords by the editorial staff upon publication. A discussion refers to a collection of user comments published as response to a specific article. An article only has one discussion, which in turn can entail any number of comments. The goal of the study is to rank comments within a singular discussion to obtain the most “featured-worthy” comments out of a specific article discussion.

The 2020 dataset contains a total of 528,973 pseudonymized comments, spanning a total of 2,015 articles from NU.nl. We limited the set by selecting only articles from three news topics, using topic labels manually assigned by the editorial staff: climate change, the 2020 US election and the COVID-19 pandemic. Other topics were relatively small in sample size. In total, the 2020 dataset contains 8, 354 featured comments. On average, a discussion consisted of 267 comments (median = 143), 4.14 of which were featured on average (median = 3). This dataset was used for the training and testing of the models, as well as the initial evaluation on unseen discussions.

The second dataset contains discussions from 2023 spanning a wider range of topics: the nitrogen issue in the Netherlands, farmer protests, the local elections, climate change and the war in Ukraine. Similar to the 2020 data, the comments were pseudonymized and include a binary variable indicating whether these were featured. This dataset contains 538, 366 comments spanning 390 articles. On average, a discussion consisted of 1, 384 comments (median = 633), with the mean featured comment count at 3.73 (median = 4). Comparing to the means of 2020 it can be observed that the activity on the platform grew over the years, resulting in a much higher average comment count per discussion, while the average number of featured comments per discussion remained stable. These 390 articles from 2023 are used in the study as evaluation to test the time robustness of our models. Not only did the activity on the platform change, the content matter of the discussions from 2023 is substantially different. A featured comment recommender should be robust to topic changes over time; it should be context insensitive, obtaining similar ranking scores on the data from 2020 and 2023.

The 2020 dataset was further split into a large set of articles for training and testing, alongside a smaller set of unseen articles for ranking and evaluation on similar content on which the models were trained. We grouped and chronologically sorted the comment data by article and split them 75%/25%. The first set (75%) contained 1, 511 articles up until October 23rd 2020. These comments were used for training and testing the classifiers. To achieve this, this dataset was further split into 80%/10%/10% generating a full training, validation and test set, respectively. The 25% set is referred to as evaluation articles (2020) in Table 1. Table 1 outlines the comment distributions in all the datasets used in this study. Thus, we work with three datasets. The first one consists of the training, testing and validation splits. The second dataset contains the unseen 2020 articles, while the third set consists of unseen 2023 articles.

**Table 1:** Data set distribution.

	**Total comments**	**Featured**
Full training	295,678	4,903
Validation	36,946	627
Test	36,911	662
95/5 training set	97,660	4,903
Evaluation articles (2020)	159,438	2,162
Evaluation articles (2023)	538,366	1,453

Table 2 summarizes the feature set, present in both 2020 and 2023 data, that we used to train and evaluate our models. Several variables were calculated out of the original data. Each comment is accompanied by delta_minutes, which equals the difference between article and comment publishing timestamp. For each comment, we calculated the word count by simply counting the number of tokens in each comment text. We used the pseudonymized user IDs to aggregate user information by grouping all comments belonging to a single user. For each user in the data, we calculated their total comment count and total featured comment count. We calculated ratio_featured by dividing the latter by their total comment count. Such ratios were also calculated for the number of replies and respect points of a user. As a last user variable, we calculated the average word count across all their comments (Table 2).

**Table 2:** Variable list: all variables used in the study.

**Var category**	**Var name**	**Description**
Comment info	Delta_minutes	Minutes between article and comment publication
	Reply_count	Absolute number of replies
	Respect_count	Absolute number of likes
	Wordcount	Number of words in the comment
User info	Total_posts_user	Total posts by user
	Featured_posts_user	Total featured posts by user
	Ratio_featured	Featured posts relative to total posts by user
	Ratio_rejected	Rejected posts relative to total posts by user
	Ratio_reply	Average reply count on posts by user
	Ratio_respect	Average number of likes on posts by user
	Avg_wordcount	Average wordcount of user
Context	Conversation similarity	Cosine similarity with mean discussion embedding
	Article similarity	Cosine similarity with article text
Content	Bag-of-Words	BoW representation of comment text
	RobBERT embedding	Mean sentence embedding extracted from finetuned model

To obtain the context variables, i.e. cosine similarities between the comments and their wider conversation and article it was commented on, we finetuned a pre-trained Dutch transformer-based language model, RobBERT (Delobelle et al., 2020). We finetuned the model on the default masked language task and trained it for 10 epochs with a batch size of 64, AdamW optimizer and a learning rate of 5*e*^−5^ (Loshchilov and Hutter, 2019). Using the SentenceTransformers package, we obtained a vector representation of each comment and article by averaging the RobBERT embeddings across all 786 dimensions (Reimers and Gurevych, 2020). The context variables were calculated following the procedure outlined by Diakopoulos (2015b). Similarity scores with the article were obtained by calculating cosine similarity between each comment and their article text. We obtained conversation similarity by calculating the mean embedding of each discussion and subsequently calculating cosine similarity between this embedding and each comment within the discussion (Diakopoulos, 2015b). While not all models included text representation of the comment, we included certain iterations with either a Bag-of-Words representation or the vector representation of the comment embedding obtained from the RobBERT model (Table 2).

**Figure 1:**
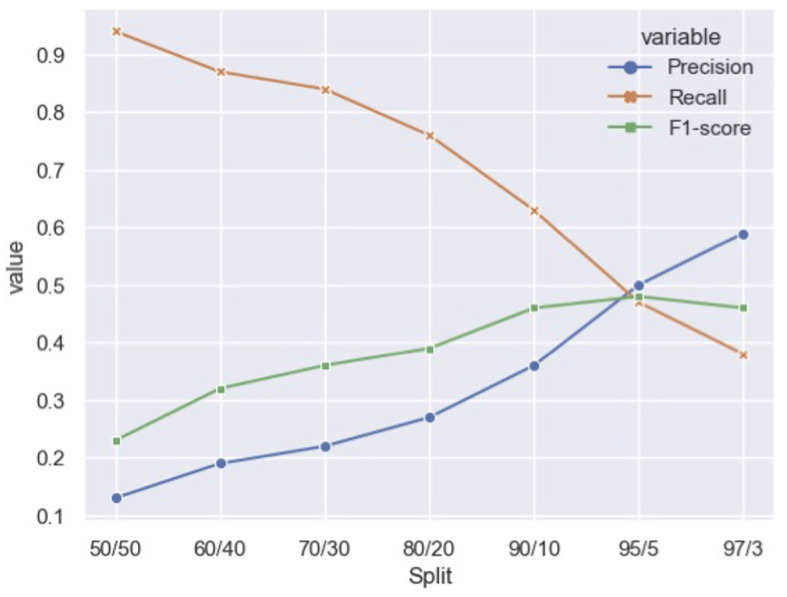
Training data splits: classification scores on validation set.

The validation set was used to calculate the optimal downsampling of non-featured comments in the training set. Excluding the text representation variables outlined in Table 2, we trained a random forest to predict if a comment was featured on seven different downsampled training sets (Figure 1). These splits include all 4, 903 featured comments found in the training set merged with a varying degree of non-featured comments. Using the scikit implementation^5^, the downsampling was performed by randomly selecting the appropriate number of non-featured comments, relative to the total number of featured comments. For example, the 50/50 ratio includes all 4, 903 featured comments along with a random selection of 4,903 non-featured comments. The ratios (Non-featured/Featured) that were tested are presented in Figure 1. To pick the best ratio, classification scores (Precision, Recall, F1-score) were calculated on the validation dataset. The 95/5 ratio, i.e. 95% non-featured comments and 5% featured comments, yielded the best result and is used as the training data henceforth. While the 95/5 ratio still remains unbalanced, the unsampled actual ratio approximates 98/2. Thus, the 95/5 training set constitutes a marked downsampling of non-featured comments.

### 2.4 Models

The upcoming paragraphs detail the models that were trained as part of this study. We have trained models without the text representation as well as a transformer-based model with purely textual input. Finally, we combined both by training two random forest models with both the non-content variables and text representation in the feature set. All models were trained on the 95/5 training set (Table 1).

#### 2.4.1 Baseline

We created a threshold-based model as baseline. Specifically, to determine whether a comment is classified as featured, the comments are ranked in descending order by the featured comment ratio of the user. Users with a ratio above 3%, the 95th-percentile, received the featured label. The intuition behind this simple baseline model is that users with a history of writing featured-worthy comments might do so in new discussions as well.

#### 2.4.2 Support Vector Machine

Using the variables described in Table 2 excluding the content category, we trained a Support Vector Machine (SVM) with the radial basis function (RBF) used in the scikit implementation.

#### 2.4.3 Random forest

We trained a random forest on the non-content variables outlined in Table 2. The standard sci-kit implementation of random forest was used and we perfomed a hyperparameter grid search. The final model has a max depth of 20, minimum samples to split a node of 5 and 1, 400 estimators.

#### 2.4.4 Text representation baseline models

While previous models were trained on the set of variables excluding the content category, we also trained a set of model exclusively on the textual input. The training data consisted of only the tokenized comment text. We employed the pre-trained transformer-based RobBERT, a Dutch language model based on the robBERTa architecture (Delobelle et al., 2020). The sequence classification RobBERT model employs a linear classification head on top of the pooled output and was trained for 10 epochs (Wolf et al., 2020). The model had a batch size of 64, AdamW optimizer and a learning rate of 5*e*^−5^. The second model trained exclusively on textual data is a bidirectional LSTM. We trained this biLSTM for 10 epochs with Adam optimizer, a batch size of 32 and binary cross-entropy. The third and final model is a Convolutional Neural Network (CNN) trained on the tokenized training texts. We trained the CNN for 10 epochs with a batch size of 32. The latter two NLP models were implemented using the Keras python library^6^. These three models represent state-of-the-art text classification models, suited for comparing the performance of models trained on our non-textual datasets (Raza et al., 2024).

#### 2.4.5 Rf_BoW & Rf_emb

The final two models combine text representation with the variables used in previously discussed classifiers. We extracted the embeddings from the RobBERT model by averaging them across all 768 dimensions using the SentenceTransformers package, resulting in a single vector per comment (Reimers et al., 2020). This vector was combined with the feature set and used to train a random forest (Rf_emb). A hyperparameter grid search was performed resulting in a final random forest model with a max depth of 100, 600 estimators and a minimum of five samples to split a node.

For the final model (RF_BoW), we represented the content of each comment by a standard Bag-of-Words approach. This method simply counts the occurrences of the tokens in each comment. We lowercased the text and removed punctuation and stopwords. We used the sci-kit implementation of Bag-of-Words and included n-grams up to three words. To reduce the size of the word set, we kept only the tokens appearing in <5% of comments, thus removing common, and less informative, words and phrases. Once again, we performed a hyperparameter grid search to train the random forest which resulted in Rf_BoW with a max depth of 20, 1, 400 estimators and a minimum of 5 samples to split a node.

### 2.5 Ranking and evaluating discussions

While the initial testing of the models is done by calculating standard classification scores, the goal of the study is to rank comments within their discussion to provide the moderator with the comments most likely to be featured based on the predictions by the model. To achieve this, we ranked comments in a discussion based on the class probability of being featured in descending order. Each discussion ranking is evaluated by calculating Normalized Discounted Cumulative Gain (NDCG) at three sizes: at 3, 5, and at 10 (Jarvelin and Kekalainen, 2000). An article has on average three featured comments, while 5 and 10 allows for the moderator to have a somewhat larger pool of options to choose from. NDCG is an often used metric to evaluate recommendation or ranking models and evaluates the top comments within the ranking, i.e. those that are shown to the moderator, in relation to the “ideal” ranking (Wang et al., 2013). In this case, the ideal ranking (Ideal Discounted Cumulative Gain, IDCG) is one that returns all correctly featured comments before showing non-featured comments within the ranking size.

NDCG is a useful metric since it takes into account the order within the ranking, meaning that comments high up in the ranking have a higher weight than those ranked lower. Therefore, models correctly ranking featured comments high in their output are rewarded, while incorrectly classified comments with a high class probability are penalized most. Scores range from 0 to 1 with a result of 1 indicating the best possible ranking. In practice, article discussions are handled one at a time. Subsequently, NDCG scores are averaged across all articles in the particular evaluation set, be it the unseen articles from the 2020 data or the recent 2023 evaluation articles. This procedure resulted in mean NDCG scores at the three ranking sizes for each trained model.

## 3 Results

### 3.1 Initial classification

The initial evaluation of the previously discussed models is done on the test set that we obtained out of the original 80/10/10 split that produced the training, validation and test sets. The latter contained 36, 911 non-featured alongside 662 featured comments published in 2020. The imbalance between featured and non-featured content illustrates the difficulty of the classification task, as merely 1.7% of the set belong to the featured class. Before moving on to ranking, the test set follows the standard procedure of a classification problem, not yet ranked by class probability. The classification scores are summarized in Table 3.

**Table 3:** Classification results on initial, unbalanced test set (1.8% featured comments).

**Model**	**Precision**	**Recall**	**F1**
Baseline	0.15	0.34	0.20
SVM	0.61	0.42	0.50
RF	0.52	0.52	0.52
RobBERT	0.17	0.29	0.21
CNN	0.10	0.24	0.14
biLSTM	0.11	0.14	0.12
Rf_BoW	0.57	0.55	0.56
Rf_emb	0.59	0.48	0.53

The baseline model achieved an F1-score of 0.20. The model lacks in precision (0.15), while achieving a slightly better recall-score of 0.34.

Our SVM model achieved the highest precision score at 0.61, although it lacked in recall (0.42) resulting in an F1-score of 0.50. RF, the random forest lacking text representation achieved a higher F1-score of 0.52 based on a balance in its precision and recall scores. Training a model purely on the textual content produced poor classification results. Compared to the baseline, our finetuned RobBERT model performed only slightly better (0.17). However, in terms of recall, the transformer-based model achieved a score of 0.29, even underperforming relative to the simple baseline model. Similarly, the CNN achieved an F1-score of 0.14, achieving the lowest precision (Table 3). The biLSTM achieved an even worse performance on the highly unbalanced test set, yielding an F1-score of 0.12. These results indicate that classifying featured comments based on text representation alone does not produce a working solution, potentially due to the fact that identical comments are not always featured.

Rf_emb was trained on the combination of the previous RF with the averaged embeddings of the comments derived from the RobBERT model. This newly obtained feature set did improve the precision-score of the original RF model by 7 percentage points (Table 3). However, this improvement was at a cost in terms of recall, achieving a recall-score of 0.48. This trade-off meant a F1-score boost of just a single percentage point.

Finally, the model Rf_BoW, which combines the RF model with Bag-of-Words text representation, achieved the highest F1-score at 0.56. RF_BoW yielded the highest recall score (0.55) and a precision score of 0.57 (Table 3).

### 3.2 Evaluation: ranking within discussions

Besides the standard classification of rare featured comments, the models ought to be able to correctly rank those featured comments in the shown set of comments. As outlined in earlier sections, we evaluated our models at different ranking sizes: 3, 5, and 10. On average, an article had 3 featured comments, while the ranking sets consisting of 5 or 10 comments give the moderator the opportunity to pick and choose. The rankings were created by sorting all comments within a discussion based on probability of belonging to the featured class. The comments with the highest probability were ranked first.

#### 3.2.1 Precision scores of rankings

Before evaluating the ranking models by calculating NDCG scores giving higher ranked comments more weight, we calculated average precision scores at sizes 3 and 5. We omitted ranking size 10 in this intermediate step due to the fact that articles with more than five comments labeled as featured are very rare. This greatly affects the precision score due to the fact that it no longer has correct comments to present. It does not affect NDCG scores in similar fashion, due to the fact that earlier ranked comments receive much more weight. After calculating the precision scores for each article, they were averaged to obtain a mean precision@3 and mean precision@5 for each presented model (Table 4).

**Table 4:** Mean Precision@3 and mean precision@5 calculated on the 2020 evaluation set.

**Model**	**Precision@3**	**Precision@5**
Baseline	0.22	0.19
SVM	0.62	0.53
RF	0.64	0.53
RobBERT	0.18	0.17
CNN	0.15	0.14
biLSTM	0.14	0.13
Rf_BoW	0.67	0.57
Rf_emb	0.31	0.26

The data used for this evaluation step was the collection of unseen 2020 articles. This set contained 471 unseen articles (159, 543 comments, 2,162 featured) with similar content matter compared to the data that were used in training and previous testing. In total, this set consisted of 351 articles on the Covid-19 pandemic, 25 on climate change and 95 on the US election in 2020.

Overall, precision decreased when the ranking size increased (Table 4). Reflecting the good performance on the initial test set, RF_BoW achieved the highest precision scores at both size 3 (0.67) and size 5 (0.57). The SVM and random forest (RF) achieved similar precision scores. The former yielded a precision of 0.62 at ranking size 3 and 0.53 at ranking size 5. RF achieved identical precision at ranking size 5, but achieved a precision of 0.64 when taking into account 3 comments with the highest class probability (Table 4).

The baseline model only taking into account the history of being often featured in the past outperformed the models exclusively trained on textual data. The baseline model achieved a precision score of 0.22 at size 3, a higher result than robBERT (0.18), CNN (0.15) and the biLSTM (0.14). Similar results were found at ranking size 5 (Table 4). However, these precision scores do not take into account the position of a comment within the ranking. To evaluate whether the discussed models achieve such correct positioning, in which the moderator first reads correctly recommended comments, we calculated NDCG scores.

#### 3.2.2 Evaluation on unseen 2020 articles: same topics as training data

Rankings were evaluated on an article basis by calculating NDCG scores at every ranking size. Subsequently, NDCG scores were averaged across all articles, producing a mean NDCG@3, 5, and 10 per model. The evaluation of the ranking capabilities of the models is threefold. First, we evaluated the models on the unseen 25% split of the 2020 dataset. This set deals with content similar to the training and testing data that we previously used. Second, we moved on from the content from 2020 and evaluated our models on unseen discussions originally published in 2023. On average, these discussions are much longer than those from 2020 and deal with a different range of topics. It is important that our models can deal with changing contexts, as the focus of news articles continuously changes. To probe the context-sensitivity, we present ranking scores per topic for both the 2020 and 2023 evaluation articles. Last, the current moderators employed at the NUjij platform evaluated the output of our best performing model in an offline, survey-style evaluation by choosing which comments to feature from a randomized list including highly ranked comments and random non-ranked comments within a random set of discussions.

On the unseen 2020 articles, the simple baseline model achieved an optimum NDCG score at ranking size 10, reaching 0.50 (Table 5). At smaller ranking sizes, the baseline model achieved lower NDCG scores. The SVM model outperformed the baseline, achieving a better ranking @3 and @5 (0.86) compared to @10 (Table 5). Subsequently, the random forest model without text representation (RF) performed better, achieving its optimum mean NDCG@3 equal to 0.89. The transformer-based RobBERT model, trained only on text representation in the form of text embedding, barely beat the baseline model (Table 5). Mimicking the poor performance on the initial test set, the NLP models yielded poor ranking results.The RobBERT model underperformed compared to the other trained classifiers, achieving an optimum NDCG score at ranking size 10 of 0.51, merely an 0.01 increase relative to the baseline model. Similarly, the CNN and biLSTM models yielded poor ranking results, even underperforming compared to the RobBERT model (Table 5).

**Table 5:** Average ranking scores calculated on unseen 2020 articles.

**Model**	**NDCG@3**	**NDCG@5**	**NDCG@10**
Baseline	0.42	0.47	0.50
SVM	0.86	0.86	0.85
RF	0.89	0.88	0.86
RobBERT	0.43	0.46	0.51
CNN	0.25	0.30	0.37
biLSTM	0.26	0.30	0.34
Rf_BoW	0.90	0.89	0.88
Rf_emb	0.71	0.72	0.72

The embeddings of the RobBERT model did not increase performance of the random forest, even decreasing the ranking scores. Rf_emb achieved a NDCG@3 of 0.71 and 0.72 for both ranking size 5 and 10. The final model, which combines the random forest with Bag-Of-Words text representation, slightly outperformed the others, achieving an optimum NDCG@3 of 0.90 and NDCG@5 of 0.89 (Table 5). This model had already achieved the optimum F1-score on the initial test set.

**Figure 2:**
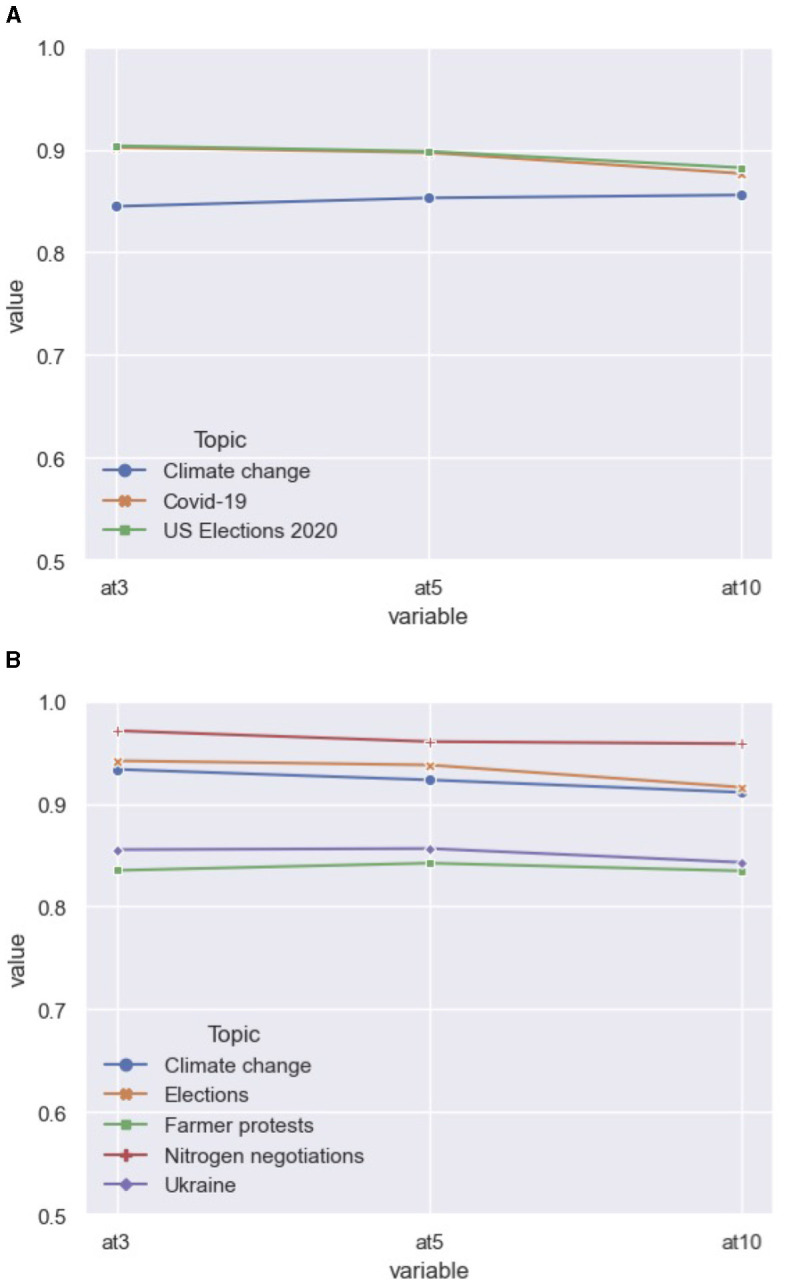
Average NDCG scores per topic by RF_BoW. **(A)** Performance on 2020 discussion topics. **(B)** Performance on 2023 discussion topics.

As context-independent ranking is the goal, we unpack the three main topics in the 2020 dataset (Figure 2A). Using the output of the best-performing model RF_BoW, we found similar ranking scores across topics. Using the Kruskal–Wallis *H*-test, we compared each NDCG@ score between the article topics.^7^ We found no significant difference between the topical groups at ranking size 3 (*H* = 0.79, *p* = 0.67), size 5 (*H* = 1.28, *p* = 0.53) and the largest ranking size 10 (*H* = 0.30, *p* = 0.86). Therefore, we conclude that our best-performing model does not perform better on any topic over the others (Figure 2A). However, the models ought to be validated on articles covering topics not found in the training and initial testing data to fully test whether the rankings work in a context-independent manner.

#### 3.2.3 Evaluation on recent data: different topics

As discussed earlier, the platform in question, Dutch NUjij, saw a stark increase in user activity recent years. Additionally, news cycles rapidly introduce novel topics to the online comment section. Ranking models supporting the content moderator ought to be able to cope with these changes in content matter and be generalizable across contexts. The goal of this second evaluation is to probe whether the models achieve similar mean NDCG scores compared to the 2020 data, as well as a topical breakdown of the results. The latter is used to analyze whether the ranking models are adequately resistant to unseen topics.

For this particular reason, we included a second dataset in the evaluation of our ranking models. This dataset contains a total of 390 unseen NUjij articles published throughout 2023. More specific, this second dataset contains 538, 366 comments, of which 1, 453 were featured by a moderator. In total, this evaluation ranked 47 articles on the topic of climate change, 112 on the farmer protests in the Netherlands, 33 on the nitrogen issue, 20 articles discussing the Dutch local elections and 35 discussions on the topic of the war in Ukraine.

**Figure 3:**
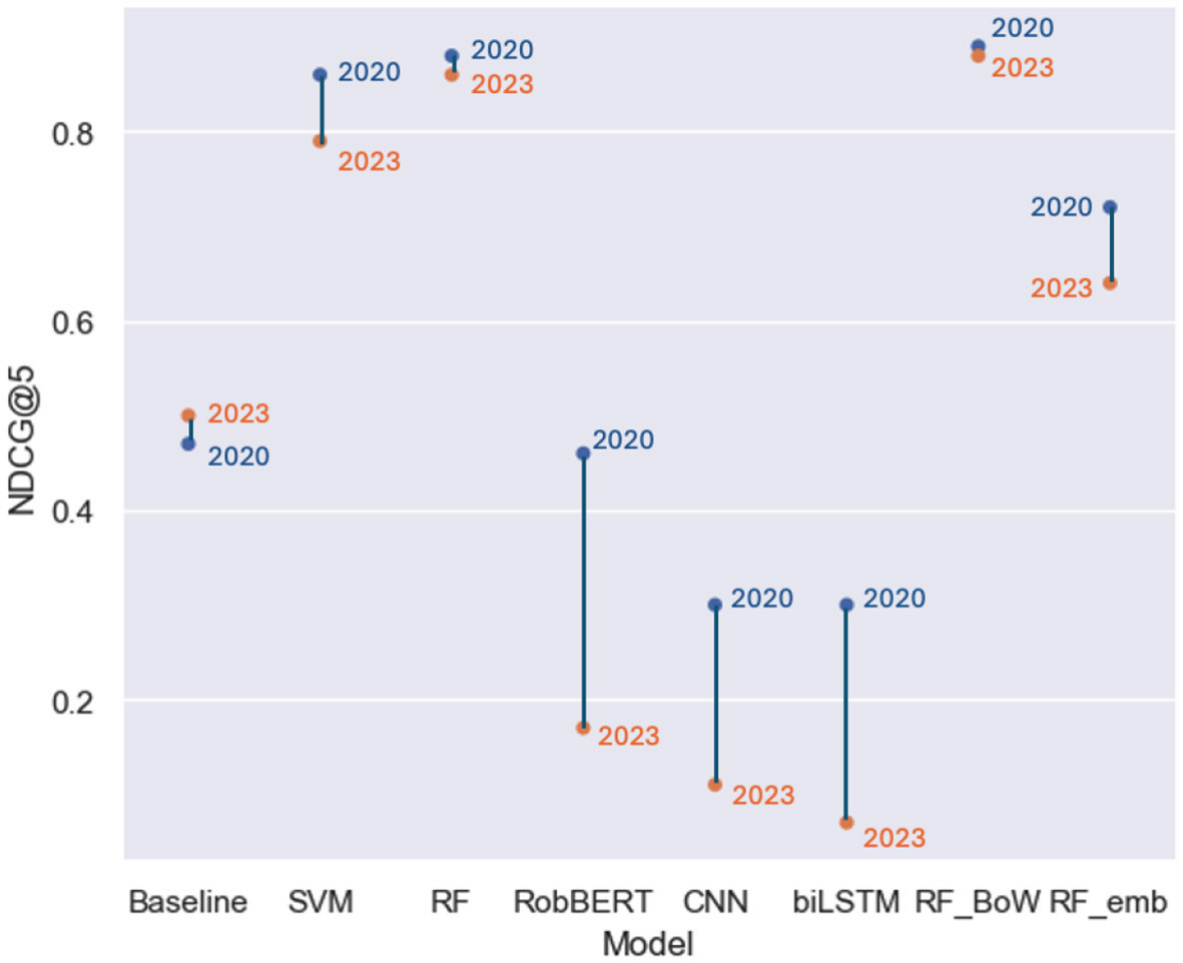
NDCG@5: Performance on evaluation sets.

Overall, the ranking output of our models was not heavily impacted by the novel data (Figure 3). Interestingly, the simple baseline model yielded improved NDCG scores on the 2023 article set. While still lacking the ranking precision of other models, all ranking sizes did experience a slight increase in NDCG score (Table 6). The SVM model experienced a relatively large decline in performance, achieving an NDCG@5 and NDCG@10 of 0.79, a decline of respectively 0.07 and 0.06. The basic random forest model (RF) achieved NDCG scores of 0.86 across the board. This result constitutes a small decline at smaller ranking sizes, while the NDCG@10 score remained equal. Our best-performing model, RF_BoW did not experience a stark decrease in performance. At ranking size 3, the model lost 0.02. At the larger ranking sizes used in the evaluation, Rf_BoW lost only 0.01 in terms of NDCG score, which still yielded the highest ranking metrics across all trained models (Table 6).

**Table 6:** Average ranking scores calculated on unseen 2023 articles.

**Model**	**NDCG@3**	**NDCG@5**	**NDCG@10**
Baseline	0.48	0.50	0.52
SVM	0.78	0.79	0.79
RF	0.86	0.86	0.86
RobBERT	0.15	0.17	0.21
CNN	0.09	0.11	0.14
biLSTM	0.05	0.07	0.10
Rf_BoW	0.88	0.88	0.87
Rf_emb	0.61	0.64	0.66

The models that did break as a result of the topical changes were those trained on textual data: the transformer-based RobBERT model, CNN and biLSTM (Figure 3). Due to their sole input being text representation, the model did not make accurate rankings when the context changed drastically in the 2023 dataset. For example, the RobBERT model experienced a decrease of 0.28 at ranking size 3, 0.29 at size 5 and even 0.30 in terms of NDCG@10 (Table 6). This drop in performance also affected RF_emb. This model combining the set of variables with the mean text embeddings from RobBERT achieved an NDCG@3 score of 0.61 on the 2023 set, a drop of 0.10. At larger ranking sizes, the model achieved a score of 0.64 and 0.66, a drop in performance of 0.08 and 0.06 respectively (Figure 3).

Once more, we took a closer look at the specific distribution of topics found in the data (Figure 2B). The scores were derived from the best-performing model, Rf_BoW. We conclude that, while all topics produced good NDCG scores, the ranking on certain topics yielded slightly better results. Using the Kruskal-Wallis test, we found significant differences between the set of topics (*H* = 29.35, *p* < 0.01). Using Dunn's test for post-hoc testing, we conclude that the ranking on the topics “Farmer protests” produced less accurate rankings, as well as the topic on the war in Ukraine compared to the articles discussing the “Nitrogen negotiations” in the Netherlands. However, even the NDCG@ scores on those particular topic are still high, hovering around those found in the 2020 data. It is the case that the other 2023 topics produced very accurate rankings, leading to the significant differences among unseen topics.

We conclude that our models, aside from those based on text embeddings, were robust against the changes within online comment sections, both originating from topical focus of the articles as well as the stark increase in activity. The best-performing model on the initial test set, RF_BoW, also achieved the highest ranking scores for both the 2020 and 2023 evaluation articles. While some topics in the more recent 2023 article set outperformed others present in the set, the ranking scores mimic those calculated on the unseen 2020 articles.

#### 3.2.4 Expert evaluation by content moderators

Moderators currently employed at NU.nl were contacted through their team supervisor and agreed to evaluate the output of the study. In total, four content moderators separately participated in the expert evaluation and validated the output of a selection of ranked online discussions from the 2020 dataset. Each individual moderator first evaluated a shared set of articles, used to calculate inter-rater agreement. Afterwards, a unique set of news articles alongside the corresponding online discussion was evaluated by each moderator to maximize the evaluation size. In total, each moderator read and evaluated the discussion of 15 articles, five shared between the four moderators and 10 unique discussions.

Using the output of Rf_BoW, a random set of unseen articles from the 2020 dataset was collected alongside the rankings made by the model. An online survey was created consisting of 30 news articles combined with a set of comments. These user comments comprised of the top ranked comments by our model (comments with class probability above 0.50 and maximum 10 comments per article), alongside an equal number of randomly selected non-ranked comments from the same online discussion. These were shuffled randomly so the moderators did not know which comments belonged to the top ranking. To replicate the real-life practice in which content moderators at NU.nl pick featured comments out of a discussion, moderators were first presented with the actual news article. Underneath, the set of comments was shown. Each comment was supplemented by information that moderators have access to in the real-life practice: the total number of previously posted and featured comments by the user, the rejection rate of the user and the number of respect points the comment received. This procedure replicates the real-life process in which comments are judged individually, while the human moderator takes the discussion context into account.

The survey question presented to the four moderators was to decide for each individual comment, within the context of the article, whether they though it was a candidate to be featured on the comment platform. The expert evaluation showed the large variation and subjectivity that this practice entails. Using the shared set of articles, we calculated a Krippendorff's alpha inter-rater agreement of 0.62. Additionally, we compared the choices made by moderators during the expert evaluation with the featured picks in the original 2020 data. We found that 42.3% of included comments that were featured in 2020 were not chosen as featured-worthy comments during the survey. These numbers indicate that the moderation practice entails a notion of subjectivity that the moderator brings to the table themselves, strengthening the concept of ranking and recommending a set of comments to choose from.

While the variation in selected comments by human moderators may pose difficulty to computational models, the expert evaluation validated the ranking performance presented in this study. In all but one article did the moderators find comments to feature among those coming from the ranking, resulting in a NDCG score of 0.83. They did not always decide on the exact same comments. Even though a large portion of subjectivity and context-specificity is involved in the process of picking featured comments, the moderators did consistently feature comments from the set produced by the model. They were more likely to feature comments which were recommended by the model (64%) compared to comments belonging to the random non-ranked set (36%).

## 4 Discussion

The presented approach differs from previous research in terms of time and topic changes as well as evaluation. Previous research mainly used artificially created test sets, while we opted to evaluate on an article basis. The latter mimics the practical setting of online content moderation and picking featured comments in particular. The lower classification scores derived from the initial evaluation on the highly unbalanced test set underscores the need to evaluate featured comment classification on data that follows the real-life distribution. Training and testing on balanced datasets, as done in most research on featured comments, produces better classification scores. However, this does not adequately portray the task of content moderation and can lead to models overestimating the number of featured-worthy comments. Moving forward, models aimed at online content moderation ought to be evaluated within the specific context that they would be used. This entails a large set of separate discussions with changing topics and participation rates. This factor was overlooked up until now in research on featured comments. It is important that models aimed at featured comments are not overfitted on the specific topics from which the training and testing data were derived.

Practically speaking, the expert evaluation underscores the importance of recommending a set of suitable comments to the human moderator as opposed to classification. While the content moderators did consistently feature comments presented within the top ranking presented to them, they did not agree with the entire set as produced by the model. Additionally, they did not always agree among themselves. Thus, presenting a selection of the most suitable comments within an online discussion allows the human moderator to apply necessary subjective and contextual judgement.

### 4.1 Robustness for time and context

The context of hybrid content moderation requires models to function across changing content matters. Furthermore, platforms evolve over time, as seen in the activity difference between the 2020 and 2023 datasets. Discussions grew larger, while featured comment counts remained stable. On top of that, moderators and users alike demand explainability of computational models used in the moderation pipeline (Ruckenstein and Turunen, 2020; Molina and Sundar, 2022). Such transparency is a prerequisite for user trust in online content moderation (Brunk et al., 2019).

Online platforms can change a lot over relatively short periods of time, an aspect of content moderation that should be taken into account when developing computational models for use in this context. For instance, our datasets from 2020 and 2023 showcased stark differences in factors such as discussion size. The average discussion in 2020 consisted of 267 comments (median = 143). However, three years later the average discussion in our dataset comprised 1, 384 comments (median = 633). While some slight variability can exist due to the topical differences and the public's interest in them, such a stark difference indicates growth in platform activity. The number of featured comments per discussion remained stable, pushing the discussions toward a larger class imbalance in regard to featured and non-featured comments. Other activity-related features that were influenced by platform growth were the average respect count of a comment, which was 3.66 in 2020 and which grew to 4.87 in 2023. Another interesting change in discussion dynamics existing in our feature set is the fact that on average, comments received fewer replies. In terms of wordcount, comments became on average shorter in 2023 compared to 2020. In 2020, the average comment counted 52 words, while we calculated an average wordcount of 40 in the 2023 dataset. While all of these discussion factors influenced our dataset and the feature set used by the classifiers, it did not strongly impact the ranking performance of our better performing models.

**Figure 4:**
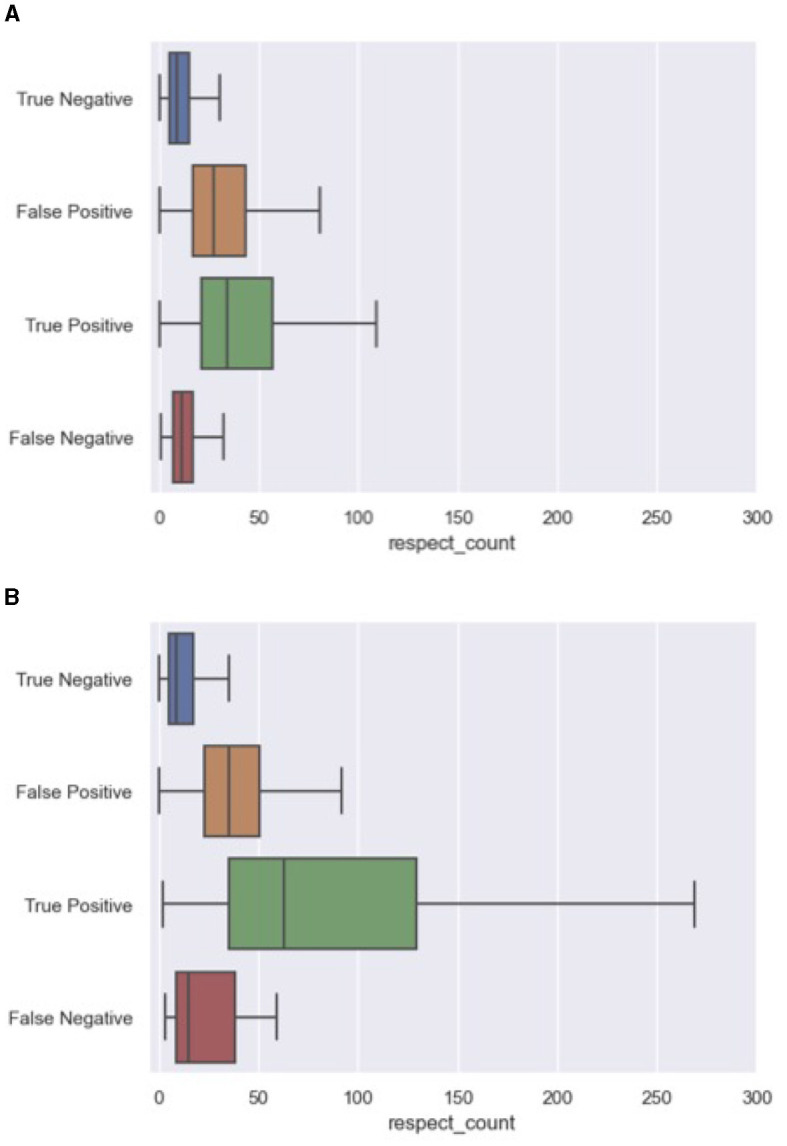
Errors on respect_count including both 2020 and 2023 articles. **(A)** Respect_count errors in 2020 articles. **(B)** Respect_count errors in 2023 articles.

**Figure 5:**
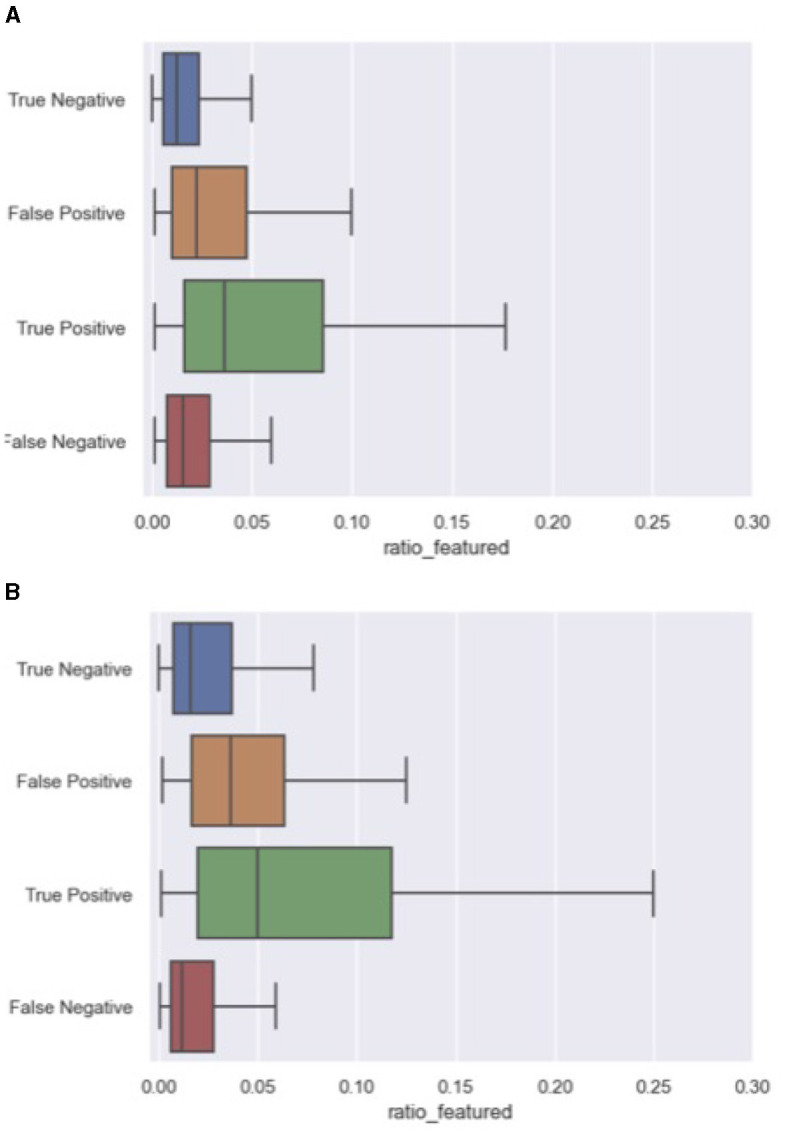
Errors on ratio_featured including both 2020 and 2023 articles. **(A)** Ratio_featured errors in 2023 articles. **(B)** Ratio_featured errors in 2023 articles.

A closer look at the correctly and incorrectly ranked comments from both the 2020 and 2023 data provide insight into the behavior of our best-performing model. More specifically, we explored whether certain features repetitively contributed to false positives (FP), and false negatives (FN). For this error analysis, we processed all unseen 2020 and 2023 articles and collected the ranked comments within each discussion at ranking size 5. The false negatives were collected from the entire discussion, since false negatives are by definition not part of the ranking. We used the python library “treeinterpreter” to collect for each prediction the feature contribution.^8^ The two most decisive variables for our model were respect_count and ratio_featured, the share of comments by a user that have been featured in the past. Interestingly, in light of the dynamics in discussion features between both datasets, the contributing factors to the incorrect predictions remained exactly the same. Figures 4, 5 outline the distribution of these variables across error categories. We conclude that the model is biased toward comments with a high number of respect points and users that have more often been featured in the past. And while these features were heavily impacted by the time-related differences between the 2020 and 2023 data, similar error patterns were found for both article sets. For example, featured comments with a low number of likes were missed, while non-featured comments with a relatively high respect count were ranked too high.

The previously presented discussion dynamics are not the only factors that have rapidly changed over time. Changing topical focus in comment sections is a given due to it following news cycles. Robustness against such fluctuation in content matter is a necessity and, as shown in earlier paragraphs, our models are capable of dealing with this aspect of online content moderation. Interestingly, however, our results indicate that, even though our best-performing model did incorporate text representation, it is not a prerequisite for achieving accurate rankings of featured comments. First and foremost, the RF model achieved slightly worse, yet similar results to its variant with Bag-of-Words text representation included in its feature set. Using the Wilcoxon signed-rank test on the NDCG scores of both models, calculated on the unseen 2020 articles, we tested whether the performance was statistically different. We found no such significant difference for ranking size 3 (*W* = 12, 124, *p* = 0.54), for size 5 (*W* = 26, 615, *p* = 0.19) and size 10 (*W* = 41, 761, *p* = 0.14). Second, the models which only used the comment text as input achieved poor results, implying that text features only offered few clues as to whether a comment was featured. Comments with identical text were sometimes featured, sometimes not. Furthermore, the labeling is not exhaustive, meaning that not all quality comments received the featured label. Only a small selection of comments were chosen per article, creating a classification task in which textually similar comments were labeled with differing classes. Combined with the topical variety found across discussions, it posed difficulty to text-based classifiers, namely RobBERT, CNN, and biLSTM (Figure 3). Thus, this result indicates the power of non-textual features. The models were robust to topical changes due to the fact that text representation only accounts for a minor share in performance.

### 4.2 The human bias of content moderation

The error analysis uncovered clear patterns in certain discussion aspects regarding featured comments. These patterns within the predictions and rankings of the classifiers arose from the already existing bias in the data. Table 7 summarizes some of the most important discussion variables, averaged for both the featured and non-featured comments in the entire 2020 dataset.

**Table 7:** Mean discussion features (calculated on 2020 data).

	**Respect_count**	**Ratio_featured**	**Wordcount**	**Ratio_rejected**	**Non-replies**
Non-featured	3	0.5%	46	23%	26%
Featured	25	6%	100	14%	35%

As already briefly mentioned in earlier sections, a bias exists in which featured comments were written by users who have been featured in the past. While this finding strengthened the hypothesis on which our baseline model was based on, it uncovered the potential human bias in which moderators favor comments of users they know write featured-worthy comments. As discussed in Section 4.1, our models mirror this behavior partly, due to the fact that ratio_featured is one of the key variables in the dataset. This bias is partly intended, the models aim to replicate the behavior of content moderators as they do not wish to change this process entirely. Featuring comments means the content presented is also endorsed by the platform, stressing the importance of the decision-making process by the moderators.

Additionally, we found that featured comments tend to be longer than the average non-featured comment (Table 7). This can naturally result of the fact that to outline featured-worthy content, a larger word count is needed, aside from the fact that very short comments are not uncommon in responses to other users. The latter are never featured, as they are part of standard discussion thread and not standalone discourse.

Other predictive patterns are comments written by users that have a lower share of rejected comments and users that tend to comment directly on the article instead of replying to comments written by other users. On average, users that received featured comments in the 2020 data wrote 36% of their comments directly to the article, while the non-featured average was 26% (Table 7). These user features paint a picture of human bias toward certain users themselves. It may be the case that such bias is wanted as a consequence of this moderation strategy. Further ranking of the output can inform the content moderator of these tendencies. For example, by presenting the featured comment ratio of users within the ranking, moderators are able to opt for comments within the ranking which were written by users who have not received a featured comment before but tick all other boxes.

All in all, the human bias in picking featured comments in online discussions on NUjij can be seen as intentional. This study made clear that certain non-content aspects of online comments, be it a user who has often been featured before or a comment with a lot of respect points, can be used to reliably rank the comments. Content moderators at the platform in question use such variables to inform or speed up their manual comment curation, whereas others like wordcount can actually be a natural prerequisite. While theoretical definitions of a high-quality comment would probably focus on content matter and presentation, training classifiers purely on textual content did not withstand the topic fluctuations in our evaluation procedure. Additionally, the contextuality caused by other features, such as the content of other featured comments and the real-world position and tone of the discussion and article topic can only be accounted for by the human moderator. For example, an obituary or a scientific news report demand a different discussion character and will influence the featured comment selection. These contextual factors cannot be integrated easily into comment datasets. The classifiers therefore incorporated this intended bias in ranking user comments in order to present a selection of comments that the moderators at the platform deem featured candidates, mimicking their past decisions. This ranking and recommendation procedure attributes the final decision-making to the human moderator, who is able to take into account the contextuality not described in actual discussion datasets.

### 4.3 Limitations and future work

The previously discussed bias in picking featured comments might be platform specific. Other platforms, including the New York Times and the Guardian have employed the moderation strategy as well. The editorial interpretation of a featured-worthy comment may differ from outlet to outlet. Future work should include a cross-platform analysis to more closely analyze the underlying comment and user distributions behind featured comments. However, the data requirements to adequately paint a full picture of online discussions are steep. Most comment datasets only entail public information. Thus, information on rejected comments would be missing. Furthermore, the aggregated user information may not be available to researchers, which forms an important component of understanding human bias in picking featured comments.

Another platform-related limitation is the language. All text used in this study was Dutch. Even though we did not test the outlined approach on data in another language, our approach, which assumes the presence of pre-labeled featured comment data and a transformer-based language model for said language, is entirely language-independent.

Future work should take a closer look at the practice of promoting good comments, as well as the human moderator making these decisions. Ethnographic fieldwork can inform researchers about the processes behind the actual featured comment choices, such as preferences for certain content or user profiles. Furthermore, such fieldwork can uncover at what times featured comment are chosen and whether it is a priority for content moderators. The context-specific nature of the moderation strategy also requires further research. Different article types or real-world setting of the story can influence the final decisions made by the human moderator, which is not described in comment datasets. A final point of focus of such future studies should be the detection of opportunities for computational models to support the human moderator within the hybrid moderation pipeline. Following the framework described in the current study, such computational approaches should focus on empowering the decision-making of the content moderator. This procedure supports the moderator and allows them to inject the contextual factors and interpretations that the computational models lack in a much needed and more efficient manner. Fieldwork is also needed to evaluate how content moderators perceive the use of computational systems within their hybrid context. Future work should ask the question whether moderators feel empowered by the use of such models and how the interaction between human moderator and computational model is perceived. While the current study did include an expert evaluation performed by a group of content moderators at the platform in question, we did not evaluate their perceptions of the hybrid moderation pipeline.

## 5 Conclusion

In this paper, we presented a classifier-based ranking system aimed at supporting the online content moderator in picking featured comments, a widespread moderation strategy. Using comment and moderation data from a Dutch news platform, we showed that combining comment data with user history and contextual relevance achieves high ranking scores. More specifically, our random forest supplemented with Bag-of-Words text representation achieved the best ranking, achieving an optimum F1-score of 0.56 in the initial testing stage. While previous research focused on classifying constructive comments validated their models only on artificially balanced test sets, we validated our models on a large set of individual articles and their discussions. This evaluation setting replicated the real-life practice of content moderation.

To test the robustness of our ranking models against changing contexts and time-related platform growth, we performed ranking evaluations on two sets of unseen articles: (1) a set of articles published in 2020 with similar content compared to the training data and, (2) a more recent set of 2023 articles with a wide range of different topics. We showed that our rankings, aside from those solely based on text embeddings, are robust against these contextual and topic factors. Next, we unpacked the individual topics in both article sets and concluded that all topics achieved high ranking scores. Furthermore, content moderators currently employed at the platform in question evaluated the output of our best-performing model. This expert evaluation yielded an NDCG score of 0.83.

We unpacked our best performing model in terms of error analysis, showing that our model favored comments from users with a history of being featured and might omit comments with a lower respect count. These findings opened up the discussion on the (intended) human bias in online content moderation, and the context-specificity that the human moderator brings to the table, a feature that cannot be extracted from comment datasets.

With our proposed and novel approach combined with model and decision-making transparency, we aim to support and empower the online comment moderator in their tasks. The human moderator plays and should play a vital role, bringing to the table contextual interpretation of an online discussion that any model lacks. With a clear and delineated role for the computational model in the hybrid moderation pipeline, we do not obscure the nuance and contextuality involved in choosing featured comments, while simultaneously improving both the experience and efficiency of online content moderation at a news platform.

## Data availability statement

The datasets presented in this study can be found in online repositories. The names of the repository/repositories and accession number(s) can be found below: https://github.com/Cwaterschoot/Featrec.

## Author contributions

CW: Writing – review & editing, Writing – original draft, Visualization, Validation, Software, Methodology, Investigation, Formal analysis, Data curation, Conceptualization. AB: Writing – review & editing, Writing – original draft, Validation, Supervision, Project administration, Methodology, Funding acquisition, Conceptualization.

## Footnotes

^1^ https://help.nytimes.com/hc/en-us/articles/115014792387-The-Comments-Section

^2^ https://www.nu.nl/nujij/5215910/nujij-veelgestelde-vragen.html

^3^ www.nu.nl

^4^ https://www.nu.nl/nujij/5215910/nujij-veelgestelde-vragen.html

^5^https://scikit-learn.org/stable/index.html, v1.2.0.

^6^https://www.tensorflow.org/guide/keras, v3.3.2.

^7^ https://docs.scipy.org/doc/scipy/reference/generated/scipy.stats.kruskal.html

^8^ https://github.com/andosa/treeinterpreter
